# Explore the Application Value of Prospective Monitoring Model in the Nursing Management of Breast Cancer Patients During Perioperative Period

**DOI:** 10.3389/fsurg.2022.850662

**Published:** 2022-02-23

**Authors:** Huan Zhang, Yu Duan, Fengming Zhou

**Affiliations:** Breast Cancer Center, Affiliated Tumor Hospital of Chongqing University, Chongqing, China

**Keywords:** breast cancer, perioperative, prospective monitoring model, value, application

## Abstract

**Purpose:**

To explore the application value of prospective monitoring model in the nursing management of breast cancer patients during perioperative period.

**Methods:**

300 perioperative breast cancer patients admitted to our hospital from January to August 2021 were randomly divided into the control group (*n* = 150) and the model group (*n* = 150). Both groups used routine nursing management, and the model group added nursing management based on a prospective monitoring model. The quality of surgical nursing, circumference of the upper limbs, and the scores of disability of arm-shoulder-hand (DASH), exerciseofself-care agencyscale (ESCA), social self-esteem scale (SSES), multidimensional fatigue symptom inventory-short form (MFSI-SF) and functional assessment of cancer therapy-breast cancer (FACT-B) were compared of the two groups.

**Results:**

Postoperatively, the quality of surgical nursing was better in the model group than in the control group (*P* < 0.05). At 3 months postoperatively, the number of cases of upper limb lymphedema was higher in both groups than before (*P* < 0.05), but there was no statistical difference between the two groups in the preoperative and 3 months postoperative comparisons (*P* > 0.05). At 3 months postoperatively, the total DASH score was higher than preoperatively in both groups, but lower in the model group than in the control group (*P* < 0.05). After nursing, the ESCA and SSES scores of each dimension were higher in both groups than before, and the model group was higher than the control group (*P* < 0.05). At 3 months postoperatively, the total MFSI-SF score was lower than preoperatively in both groups, and lower in the model group than in the control group (*P* < 0.05). At 3 months postoperatively, the FACT-B scores of each dimensions were higher in the model group than in the control group (*P* < 0.05).

**Conclusion:**

The implementation of nursing management based on a prospective monitoring model for breast cancer patients during the perioperative period has important clinical value in improving the quality of surgical nursing and improving postoperative upper limb lymphedema, upper limb function, self-care ability, social self-esteem, cancer-related fatigue symptoms, quality of life, etc.

## Introduction

Breast cancer has the highest incidence of malignant tumors among women in China and worldwide (1, 2). In China, the 5-year survival rate for breast cancer patients is about 73%, much lower than the 90% in the United States. The combination of surgery, radiation and chemotherapy treatment creates favorable conditions for saving the lives of breast cancer patients and prolonging their postoperative survival. However, the treatment is prone to several complications, such as upper limb lymphedema (3), upper limb dysfunction (4), subcutaneous effusion (5), flap necrosis (6), cancer-related fatigue (7), pain syndrome (8), Toxic side effects during radiotherapy and chemotherapy (9), anxiety and depression (10), and sleep disorders (11). All of these complications can seriously impair the physical, functional, emotional and family/social health of patients, which in turn affects the postoperative recovery process and the quality of postoperative survival. For this reason, while clinically providing advanced surgical techniques for breast cancer patients, it is also necessary to focus on the postoperative rehabilitation.

Combined with previous studies, our rehabilitation care for perioperative breast cancer patients lacks individual relevance and overall predictability. On the one hand, the health education for patients by medical and nursing staff is too mechanical and formalized, without paying attention to the real feelings and needs of each patient from a humanistic perspective, resulting in patients' deviation or disinterest in the content of the education, thus failing to achieve the purpose of cultivating patients' health self-care ability. On the other hand, the focus of medical staff's care is to solve the problems that patients have already shown. The lack of prospective intervention for possible complications or psychological problems is not conducive to the resolution of postoperative physical and mental problems. A new, intuitive, and comprehensive humanized nursing management program is yet to be implemented.

The prospective detection model is a set of clinical care management protocols proposed by Stout et al. (12) for the perioperative and Post-discharge follow-up phases of breast cancer. In previous studies abroad, it has the roles of providing health education guidance, monitoring breast cancer treatment-related physical and mental problems and dysfunction, identifying early injuries, introducing means of rehabilitation interventions when determining impairments, and promoting patients' health self-care behaviors. In recent years, our department has introduced a nursing management program based on a prospective monitoring model into the nursing management of perioperative breast cancer patients, and observed the impact of the prospective monitoring model on the postoperative recovery of breast cancer patients in China, in order to provide a favorable reference for the selection of a nursing model for perioperative breast cancer patients in China.

## Materials and Methods

### Research Object

Three hundred perioperative breast cancer patients admitted to our hospital from January to August of 2021 were selected. Inclusion criteria: age ≥20 years; belonged to primary breast cancer; first diagnosis of breast cancer by pathological examination or imaging techniques; clinical stage 0~III; single lesion without lymph node or distant metastasis; those who intended to be treated by modified radical surgery or breast-conserving surgery in our hospital; those who communicated well and could cooperate with the study; those who voluntarily signed the informed consent form. Exclusion criteria: combination of other malignant tumors or breast cancer caused by metastasis from other malignant tumors; combination of upper limb disability; combination of cardiogenic, nephrogenic or dystrophic edema; contraindication to surgery; pregnancy or lactation; combination of diabetes mellitus, immune disorders or severe liver diseases; severe intellectual deficiency, mental illness or cognitive impairment. Patients who met the inclusion criteria were randomly and equally divided into the control group (*n* = 150), and the model group (*n* = 150). Comparing the general conditions of age, menstruation, and type of surgery between the two groups, there were no statistical differences and were comparable (*P* > 0.05). As shown in Table 1.

**Table 1:** Comparison of general conditions of two groups.

**Itmes**	**Control group** **(*n* = 150)**	**Model group (*n* = 150)**	***t*** **/χ^2^ value**	***P*** **value**
Age (years old)	45.26 ± 6.02	44.98 ± 5.89	0.407	0.684
BMI (kg/m^2^)	22.89 ± 1.54	22.78 ± 1.62	0.603	0.547
Years of education (years)	12.54 ± 1.56	12.60 ± 1.55	0.334	0.739
Menopause or not (%)			0.484	0.487
Yes	79 (52.67)	85 (56.67)		
No	71 (47.33)	65 (43.33)		
Location of onset (%)			0.120	0.729
Left	77 (51.33)	74 (49.33)		
Right	73 (48.67)	76 (50.67)		
Pathological type (%)			0.140	0.708
Invasive cancer	102 (68.00)	105 (70.00)		
Non-invasive cancer	48 (32.00)	45 (30.00)		
Clinical stage (%)			1.113	0.774
0	12 (8.00)	10 (6.67)		
I	64 (42.67)	57 (38.00)		
II	44 (29.33)	50 (33.33)		
III	30 (20.00)	33 (22.00)		
Surgery type (%)			0.133	0.715
Modified radical mastectomy	97 (64.67)	100 (66.67)		
Breast conservation	53 (35.33)	50 (33.33)		

### Research Methods

Routine nursing management for the control group. That was: preoperative stage: routine preoperative preparations (such as skin preparation, drug preparation, preoperative examination, etc.); reminded to start fasting and drinking at 22:00 on the night preoperatively; relieved patients' preoperative anxiety, etc. Early postoperative stage: wound and drainage tube care, elevation and braking of the affected limb, distribution of case management manuals, instruction on rehabilitation of the affected limb and discharge precautions, etc. Follow-up stage: patients were followed up by telephone 3 days after discharge. The follow-up visit includes answering patients' concerns, instructing them on the methods of living food, pipe maintenance and rehabilitation training, and helping them to arrange matters related to hospital admission and return to hospital for review.

The model group was applied routine nursing management + nursing management based on a prospective monitoring model. This was:

Preoperative stage: The investigators used an assessment tool to conduct a baseline assessment of the patients and, based on the assessment, provided health education on preoperative and postoperative care plans before surgery. Patients were instructed to learn skills and methods of injury recognition, self-monitoring, self-management, and health promotion before surgery. Specifically: ① Preoperatively, organizing patients to watch breast cancer health education videos and distributing case management manuals for learning about surgery, treatment-related knowledge, procedures and precautions; ② Individualized psychological care for the patient's psychological state was carried out, such as instructing the patient to relieve psychological pressure through relaxation training or meditation; ③ Instructing patients in postoperative rehabilitation nursing methods, such as raising and braking the upper limbs, and informing patients about the methods of ankle pump exercise, movement and functional exercise of the affected limb; ④ Guiding patients to self-care nursing methods for postoperative wounds and drainage tubes to prevent complications; ⑤ Guiding patients to self-identify and self-detect possible precursor symptoms, and informing patients of ways to avoid future risks, such as avoiding the use of affected limbs to measure blood pressure, blood draw, and infusion to prevent lymphedema; ⑥ Analysis and prevention of various problems that might occur intraoperatively by means of quality control management methods or failure mode and effect analysis (13). For example, we strictly checked the instruments and equipment in the operating room before surgery to ensure their normalcy, sterility and integrity, and completely recorded the use and handling of intraoperative items.

Early postoperative stage: that was the period of time from the end of surgery to discharge. Patients' physical and mental status was reassessed by the researcher and self-assessed at any time by the patients themselves. Based on the assessment results, the patient's early rehabilitation training components were again developed and directed. Specifically: ① On the day after surgery, guiding patients to exercise the ankle pump to promote blood and lymphatic return to the lower extremities; ② On the 1st postoperative day, patients underwent aerobic exercise and health care providers again provided health education on postoperative care, exercise and functional exercise, and nutrition; ③ Conducting a health talk on breast cancer-related knowledge every Thursday to instruct patients on damage recognition and self-management methods; ④ On the day of discharge, re-guidance Post-discharge motor function training, risk identification, injury management, psychological counseling, and self-monitoring for patients and their families; ⑤ Baseline data testing was repeated during this period, and individualized intervention protocols were initiated once patients' self-assessment reports or researcher monitoring identified physical impairment (for example, when the arm circumference difference of the same measurement point of the affected and healthy limb was >2.0 cm, or when there was obvious pain or limited mobility).

Post-discharge continuous monitoring stage: nn the basis of routine return to hospital and review follow-up, the researchers assessed the physical and mental status of patients at 1, 2 and 3 months after surgery, and patients self-assessed at any time. Based on the assessment results, the researchers provided health education to patients on self-monitoring and self-management, and taught them injury recognition (e.g., early detection of related sequelae) and health promotion (e.g., methods of functional rehabilitation of the affected limb, maintaining healthy lifestyle behaviors) skills. At follow-up, patients were assessed for recovery of the affected limb, cause-related fatigue, occurrence of postoperative complications, quality of life, and self-care ability. Individualized interventions were initiated if changes were monitored during this period compared to the preoperative period, and if not, follow-up was continued after a 1-month interval.

### Observation Index

Quality of surgical nursing: postoperatively, the quality of surgical nursing in both groups was assessed by our own “Surgical Nursing Care Quality Assessment Form”. The Cronbach's α coefficient was 0.735. It contained 6 dimensions: material management, aseptic situation, nursing records, health education, basic nursing, knowledge assessment. Each item was scored from 0 to 100, and the score was positively correlated with the quality of surgical nursing.

Upper limb circumference: Preoperatively and 3 months postoperatively, upper limb circumference was measured in both groups to assess the occurrence of upper limb lymphedema. Upper limb lymphedema was diagnosed if the difference in arm circumference between the same measurement point on the healthy and the affected side was >2.0 cm.

Disability of arm-shoulder-hand (DASH) score: The functional recovery of the affected limbs in both groups was assessed by DASH preoperatively and 3 months postoperatively. It contained 2 parts, A (23 items) and B (7 items). Each item was scored 1–5, total score = (A+B total score −30) /1.2. Total score 0~100 represented normal~extremely restricted upper limb function.

Exercise of self-care agency scale (ESCA) score: Before and after nursing, the self-care ability of both groups was assessed by ESCA. It included 4 dimensions: health knowledge level (0–68 scores), self-concept (0–32 scores), self-responsibility (0–24 scores) self-nursing skills (0–48 scores). Scores were directly proportional to self-care ability.

Social self-esteem scale (SSES) scores: Before and after care, social self-esteem was assessed by SSES in both groups. It included 20 items in 3 dimensions: social self-esteem, behavioral self-esteem, and appearance self-esteem. A total of 100 points were scored, and the scores were proportional to the level of self-esteem.

Multidimensional fatigue symptom inventory-short form (MFSI-SF) score: Preoperatively and 3 months postoperatively, the degree of cancer-caused fatigue was assessed by MFSI-SF in both groups. It contained 5 dimensions: physical fatigue (0–24 scores), mental fatigue (0–20 scores), emotional fatigue (0–20 scores), general fatigue (0–20 scores), and vitality (0–24 scores). The total score was the total score of the first 4 dimensions–vitality score. The total score was proportional to the level of fatigue.

Functional assessment of cancer therapy-breast cancer (FACT-B) score: 3 months postoperatively, the quality of life was assessed by FACT-B in both groups. It included 5 dimensions: somatic condition (0–28 scores), functional condition (0–28 scores), emotional condition (0–24 scores), social/family condition (0–28 scores), and additional concerns (0–36 scores). The score was directly proportional to the quality of life.

### Statistical Methods

SPSS 22.0 software was applied, and the measurement data were expressed as mean ± standard deviation and compared by *t*-test. Count data were expressed as ratios, and the χ^2^ test was used for comparison. *P* < 0.05 was considered statistically significant.

## Results

### Comparison of the Quality of Surgical Nursing of Two Groups

**Figure 1:**
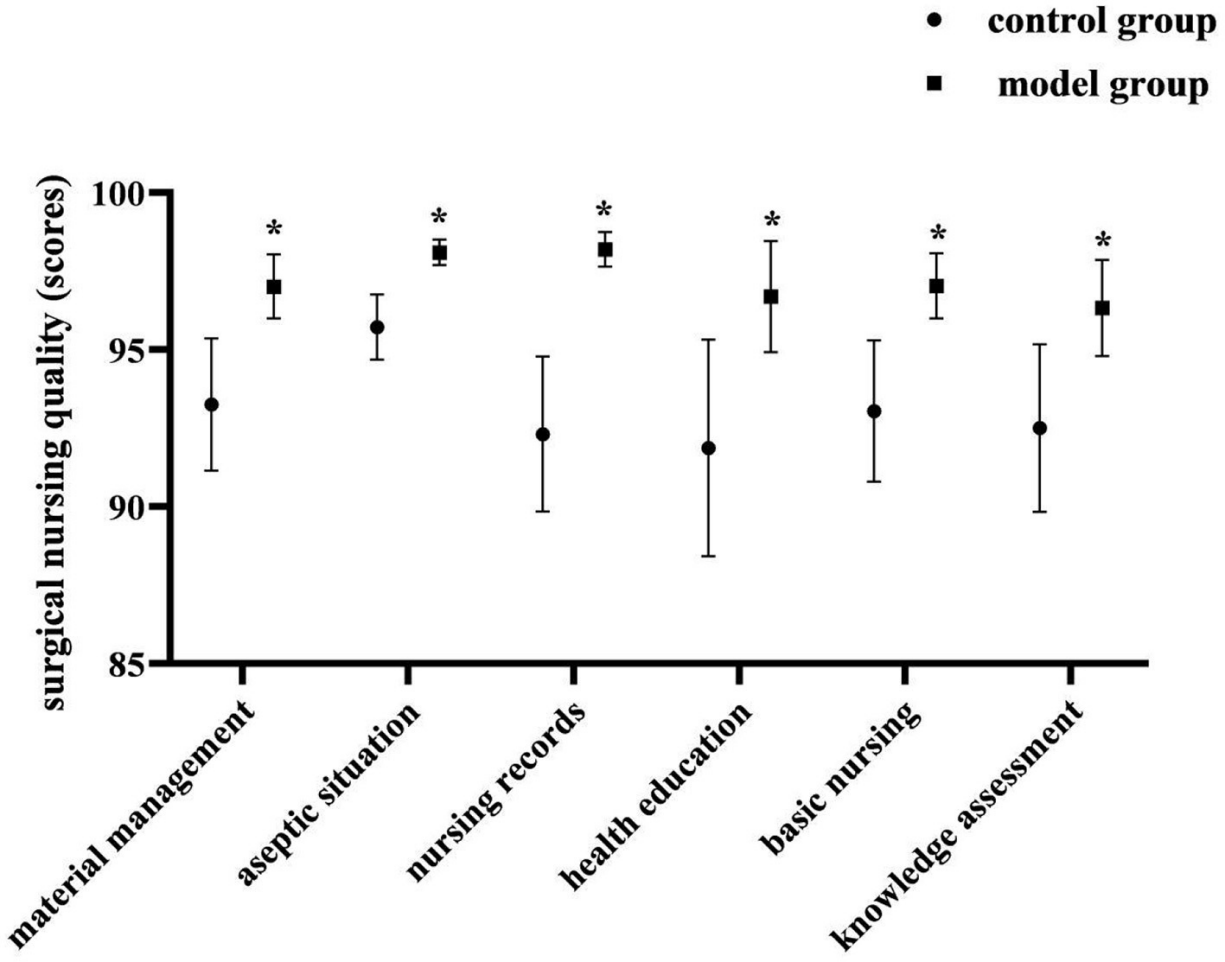
Comparison of the quality of surgical nursing of two groups. Compared with the control group in the same dimension, *P < 0.05.

Postoperatively, the model group had better quality of nursing scores than the control group on material management, aseptic situation, nursing records, health education, basic nursing, knowledge assessment (*P* < 0.05). As shown in Figure 1.

### Comparison of Upper Limb Lymphedema of Two Groups

**Figure 2:**
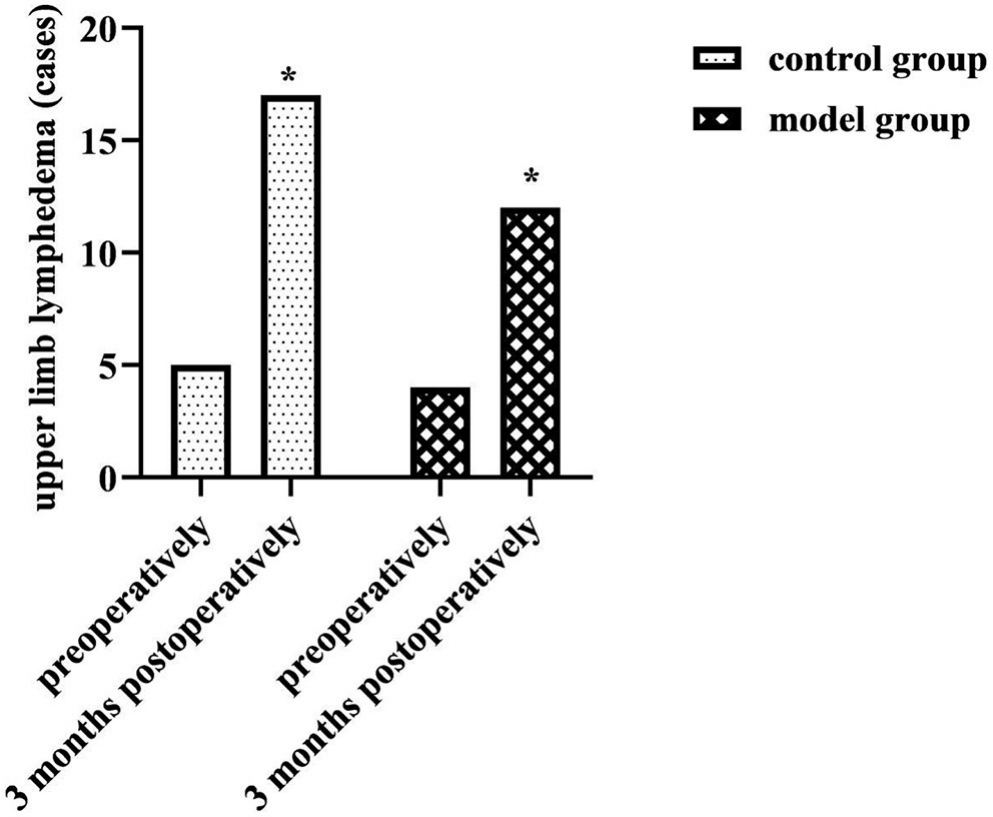
Comparison of upper limb lymphedema of two groups. Compared with the same group preoperatively, *P < 0.05.

At 3 months postoperatively, the number of cases of upper limb lymphedema was higher in both groups than before (*P* < 0.05), but there was no statistical difference between the two groups in the preoperative and 3 months postoperative comparisons (*P* > 0.05). As shown in Figure 2.

### Comparison of Total DASH Scores of Two Groups

**Figure 3:**
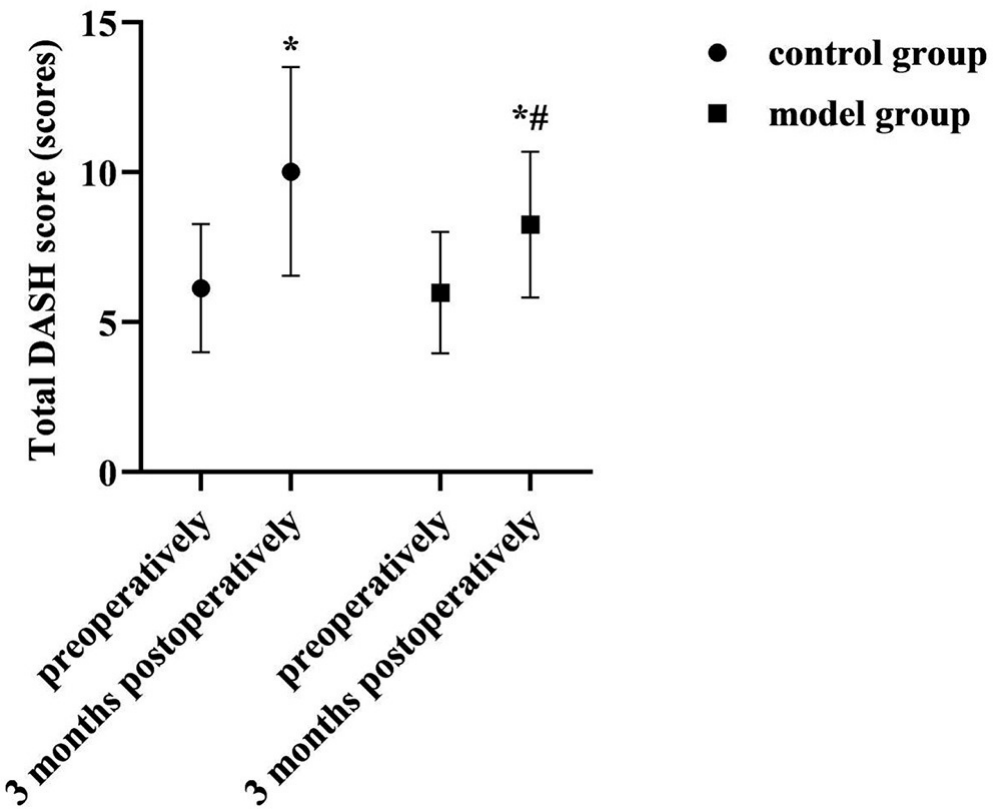
Comparison of total DASH scores of two groups. Compared with the same group preoperatively, *P < 0.05; compared with the control group 3 months postoperatively, ^#^*P* < 0.05.

At 3 months postoperatively, the total DASH score was higher than preoperatively in both groups, but lower in the model group than in the control group (*P* < 0.05). As shown in Figure 3.

### Comparison of ESCA Scores of Each Dimension of Two Groups

**Figure 4:**
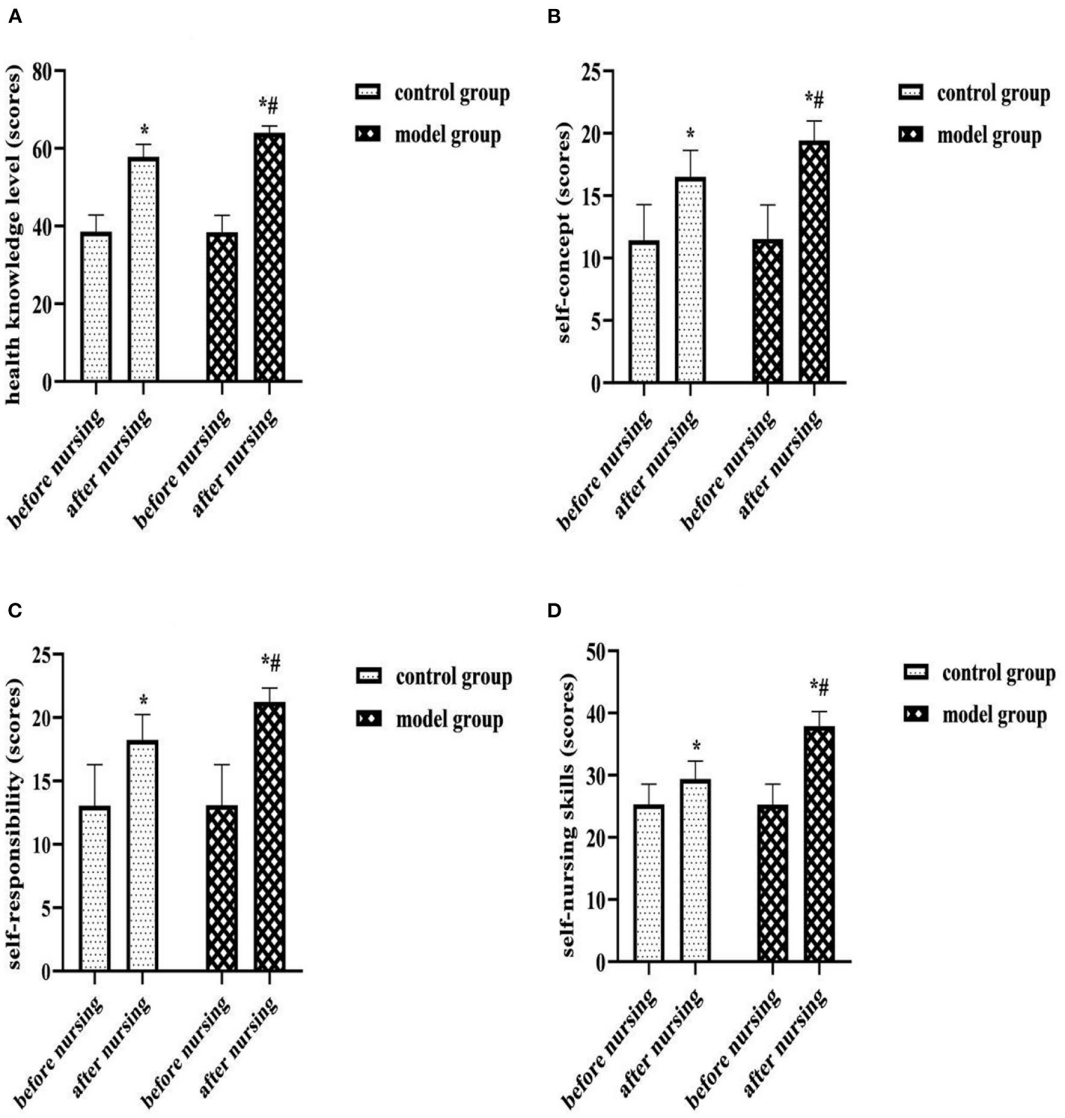
**(A)** Health knowledge level (scores). **(B)** Self-concept (scores). **(C)** Self-responsibility (scores). **(D)** Self-nursing skills (scores). Comparison of ESCA scores of each dimension of two groups. Compared with the same group before nursing, *P < 0.05; compared with the control group after nursing, ^#^*P* < 0.05.

After nursing, the ESCA scores of each dimension were higher in both groups than before, and the model group was higher than the control group (*P* < 0.05). As shown in Figure 4.

### Comparison of SSES Scores of Each Dimension of Two Groups

**Figure 5:**
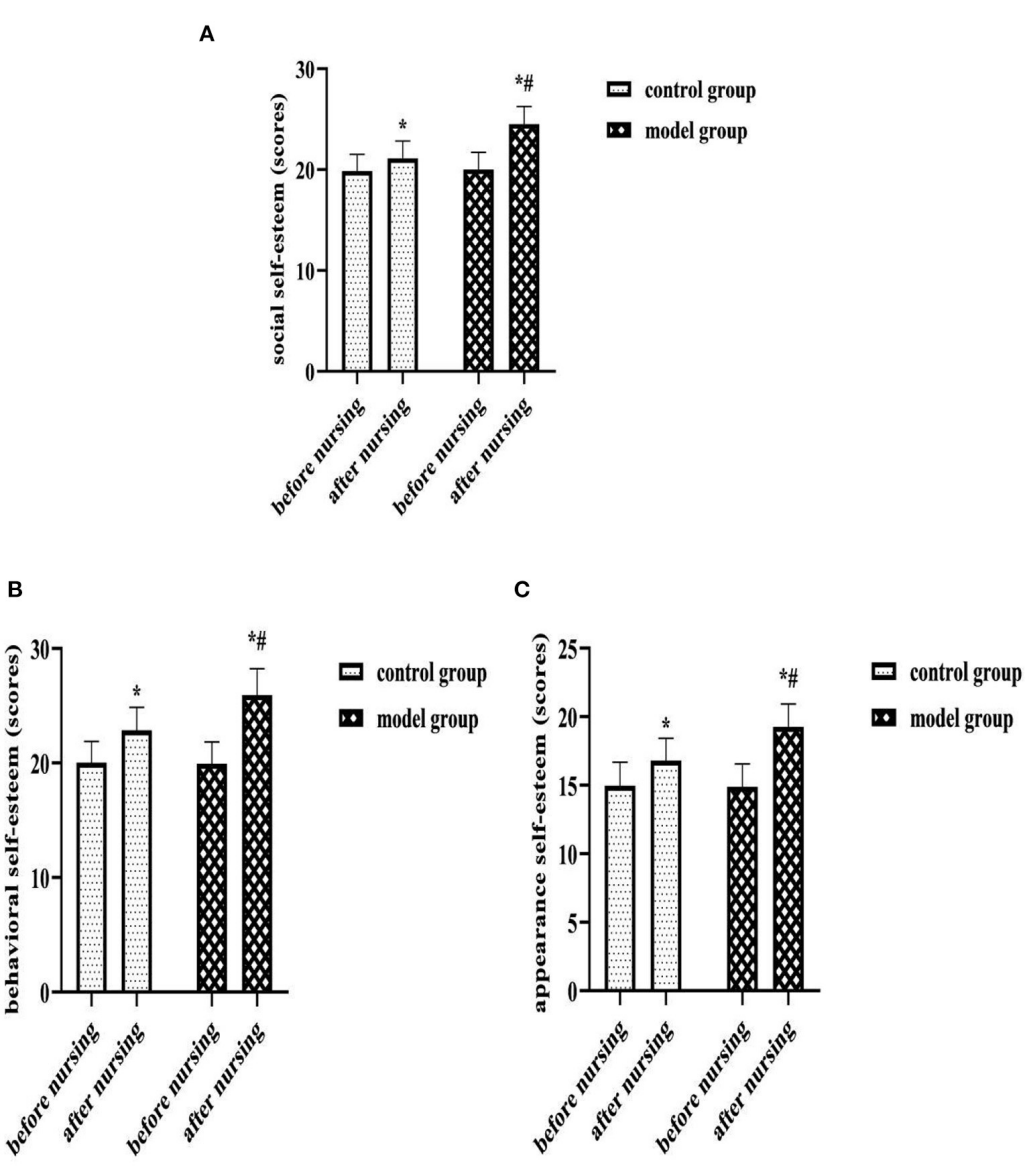
**(A)** Social self-esteem (scores). **(B)** Behavioral self-esteem (scores). **(C)** Appearance self-esteem (scores). Comparison of SSES scores of each dimension of two groups. Compared with the same group before nursing, ^*^P < 0.05; compared with the control group after nursing, ^#^*P* < 0.05.

After nursing, the SSES scores of each dimension were higher in both groups than before, and the model group was higher than the control group (*P* < 0.05). As shown in Figure 5.

### Comparison of Total MFSI-SF Scores of Two Groups

**Figure 6:**
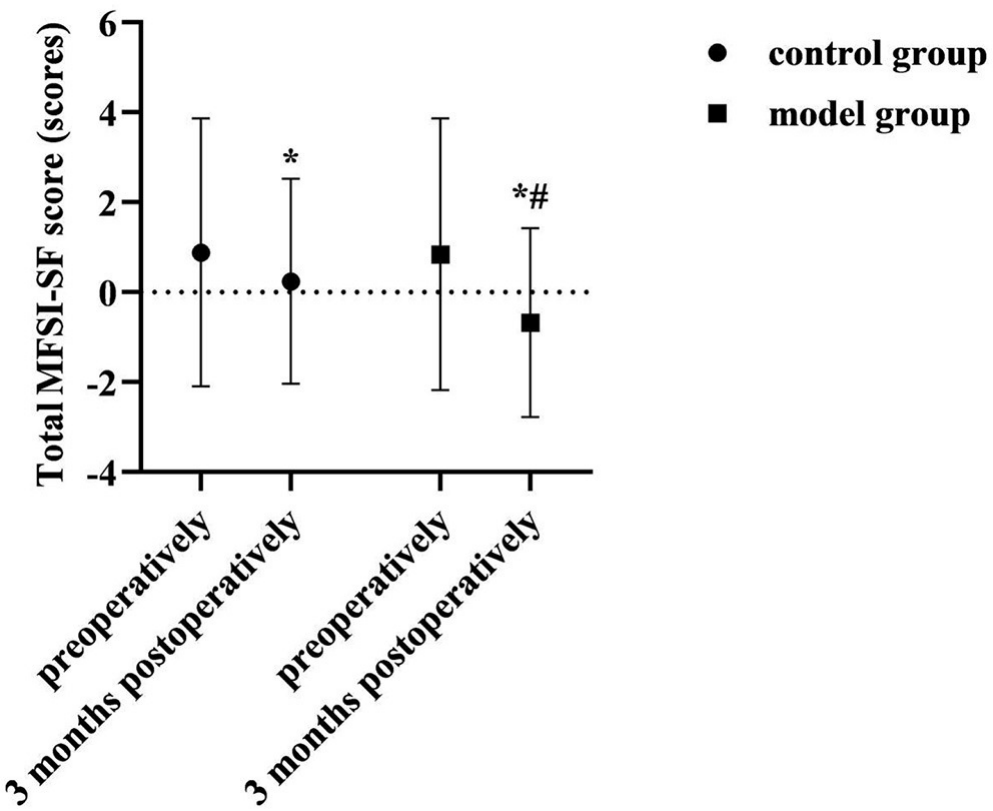
Comparison of total MFSI-SF scores of two groups. Compared with the same group preoperatively, *P < 0.05; compared with the control group 3 months postoperatively, ^#^*P* < 0.05.

At 3 months postoperatively, the total MFSI-SF score was lower than preoperatively in both groups, and lower in the model group than in the control group (*P* < 0.05). As shown in Figure 6.

### Comparison of FACT-B Scores of Each Dimension of Two Groups

**Figure 7:**
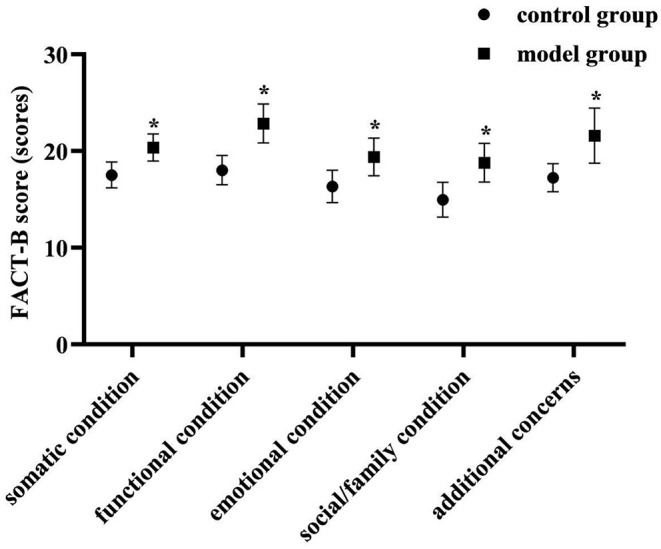
Comparison of FACT-B scores of each dimension of two groups. Compared with the control group in the same dimension, *P < 0.05.

At 3 months postoperatively, the FACT-B scores of each dimension were higher in the model group than in the control group (*P* < 0.05). As shown in Figure 7.

## Discussion

Surgery is an indispensable part of the comprehensive treatment of breast cancer, and high-quality surgical care is of great significance to increase the success rate of surgery and prolong the survival rate of patients. In the results of this study, the quality of surgical nursing was better in the model group than in the control group (*P* < 0.05). Analyzing its reasons, this study implements a nursing management program based on a prospective monitoring model. Through prospective intervention methods such as quality control management or failure mode effect analysis, it analyzes the causes of various problems and loopholes that may occur during surgery under the conventional nursing mode, and proposes appropriate and effective intervention plans and improvements accordingly. Therefore, the purpose of significantly improving the quality of surgical care can be achieved.

Upper extremity lymphedema is one of the most common complications during the 3 months to 3 years after breast cancer surgery (14). Statistically, its incidence could increase from 5 to 11% at 3–6 months postoperatively and up to 75% at 2 years postoperatively (15). It can cause chronic swelling, pain, numbness, dysfunction and other symptoms in the affected limb, subsequently leading to a series of physical, psychological and social problems (16). The incidence of upper limb lymphedema in the control group (3.33%) and model group (2.67%) preoperatively in this study was similar to previous statistics. At 3 months postoperatively, the incidence of lymphedema was higher in both the control group (11.33%) and the model group (8%) than preoperatively (*P* < 0.05), but there was no statistical difference between the two groups (*P* > 0.05). This suggested that the use of a prospective monitoring model for breast perioperative patients was no different from conventional care in preventing postoperative upper limb lymphedema. This might be related to the occult of the onset of upper limb lymphedema as a chronic complication (17). Moreover, the disease mostly occurs after 3 months postoperatively, while the postoperative observation period in this study was intercepted only up to 3 months postoperatively. Therefore, the results of this study cannot prove the long-term effect of the prospective monitoring model in the prevention and treatment of upper limb lymphedema.

Upper limb dysfunction is another common complication of breast cancer surgery. Its onset is associated with inadequate execution or inappropriate modalities of postoperative functional training of patients, inadequate education or inadequate expertise of health care professionals. In the results of this study, at 3 months postoperatively, the total DASH score was higher than preoperatively in both groups, but lower in the model group than in the control group (*P* < 0.05); After nursing, the ESCA scores of each dimension were higher in both groups than before, and the model group was higher than the control group (*P* < 0.05). Analyzing the reasons for this, we provided repeated rehabilitation training instruction and injury recognition, self-monitoring and self-management education during the preoperative, early postoperative and Post-discharge continuous monitoring stages in the care management of the model group. This not only helped to meet patients' needs for knowledge about breast cancer rehabilitation and strengthen their understanding and memory of the knowledge, but also helped to promote patients' health self-care behaviors and implementation. Moreover, continuous monitoring management and individualized intervention during the follow-up period after discharge can effectively ensure the integrity and continuity of postoperative functional training of patients, and play a certain role in supervising and regulating patients' health self-care behaviors. Therefore, the implementation of nursing management based on the prospective monitoring model is very beneficial to promote the rehabilitation of the affected limb function and the self-care ability of patients after breast cancer surgery.

Self-esteem is the result of self-evaluation and social comparison triggered by social situations, social self-esteem is a self-maintenance mechanism for individuals under external pressure, and a high social self-esteem state can effectively isolate individuals from stress in the environment (18). Based on the dual pressure of life threatening and loss of female physical characteristics, breast cancer surgery patients have more sensitive internal indicators and lower self-esteem levels compared to patients with other malignancies (19). After care in this study, patients in the model group had significantly higher social, behavioral, and appearance self-esteem levels than the control group (*P* < 0.05). Analyzing the reasons, the application of the prospective monitoring model in this group, the researchers' interval assessment of patients' physical and mental status could identify their potential negative emotional threats and psychiatric treatment needs through their behavioral responses in a timely manner, and provided positive psychological counseling accordingly, which could help patients relieve psychological stress, correct disease perceptions, establish treatment concepts, and rebuild their self-image in a timely manner. As a result, it was beneficial to restore the patient's level of social self-esteem.

Cancer-related fatigue is a persistent subjective feeling of fatigue and lack of energy associated with tumors or antineoplastic treatment (20). It can affect many aspects of patients, such as body, emotion, function, cognition and social interaction, etc., so it is also closely related to the decline of the patient's quality of life (21). At 3 months postoperatively in this study, the total MFSI-SF score was lower than preoperatively in both groups, and lower in the model group than in the control group (*P* < 0.05). At 3 months postoperatively, the FACT-B scores of each dimensions were higher in the model group than in the control group (*P* < 0.05). This suggested that the implementation of care management based on a prospective monitoring model contributed to the improvement of fatigue symptoms and quality of life in patients after breast cancer surgery. After breast cancer surgery, the inevitable decrease in self-care ability and negative psychological status can cause cancer-related fatigue symptoms. In this study, the monitoring and positive guidance of patients' physical and mental status were emphasized in the preoperative, early postoperative and Post-discharge continuous monitoring stages. Among them, the method of instructing patients in relaxation training and aerobic exercise training helped to reduce the level of physical fatigue of patients; the method of instructing patients to meditate helped to reduce the level of mental fatigue of patients. And helping patients learned self-monitoring and self-management was helpful for timely individualized intervention plans when abnormal physical and mental states were found. Thus, the implementation of nursing management based on a prospective monitoring model for perioperative breast cancer patients is an effective way to reduce cancer-related fatigue and improve the quality of life of patients after surgery.

## Conclusion

Supportive care is an extremely important part of the rehabilitation process for breast cancer patients. The application of the prospective monitoring model of this study in breast cancer patients during perioperative period could effectively address multiple issues, including improving the quality of surgical nursing for breast cancer patients, protecting musculoskeletal health after surgery, promoting patients' health self-care behaviors and abilities, rebuilding social self-esteem and self-image, reducing cancer-caused fatigue symptoms, and improving quality of life.

## Data Availability Statement

The original contributions presented in the study are included in the article/supplementary material, further inquiries can be directed to the corresponding author.

## Ethics Statement

The studies involving human participants were reviewed and approved by the Ethics Committee of the Affiliated Tumor Hospital of Chongqing University. The patients/participants provided their written informed consent to participate in this study.

## Author Contributions

All authors of this study have made equal contributions, including research design, result testing, data statistics, and paper writing.

## Funding

Decision Consultation and Management Innovation Project of Shapingba District, Chongqing, Jcd202118.

## Conflict of Interest

The authors declare that the research was conducted in the absence of any commercial or financial relationships that could be construed as a potential conflict of interest.

## Publisher's Note

All claims expressed in this article are solely those of the authors and do not necessarily represent those of their affiliated organizations, or those of the publisher, the editors and the reviewers. Any product that may be evaluated in this article, or claim that may be made by its manufacturer, is not guaranteed or endorsed by the publisher.
